# Relationship between history of coronary heart disease at dialysis initiation and onset of events associated with heart disease: a propensity-matched analysis of a prospective cohort study

**DOI:** 10.1186/s12882-017-0495-8

**Published:** 2017-02-28

**Authors:** Daijo Inaguma, Shigehisa Koide, Kazuo Takahashi, Hiroki Hayashi, Midori Hasegawa, Yukio Yuzawa

**Affiliations:** 1 Department of Nephrology, Fujita Health University School of Medicine, Toyoake, Aichi Japan; 2 The Aichi Cohort Study of Prognosis in Patients Newly Initiated Into Dialysis (AICOPP) Group, Aichi, Japan

**Keywords:** Chronic kidney disease, Coronary heart disease, Dialysis initiation, Cardiovascular disease, Mortality

## Abstract

**Background:**

Chronic kidney disease (CKD) is an independent risk factor for cardiovascular disease (CVD) events, and a number of reports have shown a relationship between CKD and CVD in pre-dialysis or maintenance dialysis patients. However, few studies have reported serial observations during dialysis initiation and maintenance. Therefore, we examined whether the incidence of heart disease events differed between CKD patients with and without a history of coronary heart disease (CHD) at dialysis initiation.

**Methods:**

The subjects were patients in the 17 centers participating in the Aichi Cohort Study of Prognosis in Patients Newly Initiated into Dialysis (AICOPP) from October 2011 to September 2013. We excluded nine patients whose outcomes were unknown, as determined by a survey conducted at the end of March 2015. Thus, we enrolled 1,515 subjects into the study. We classified patients into 2 groups according to the history of CHD (i.e., a CHD group and a non-CHD group). Propensity scores (PS) represented the probability of being assigned to a group with or without a history of CHD. Onset of heart disease events and associated mortality and all-cause mortality were compared in PS-matched patients by using the log-rank test for Kaplan-Meier curves. Factors contributing to heart disease events were examined using stepwise multivariate Cox proportional hazards analysis.

**Results:**

There were 254 patients in each group after PS-matching. During observation, heart disease events occurred in 85 patients (33.5%) in the CHD group and 48 (18.9%) patients in the non-CHD group. The incidence was significantly higher in the CHD group (*p* < 0.0001). The CHD group was associated with higher incidence of heart disease events (vs. the non-CHD group, hazard ratio = 1.750, 95% confidence interval = 1.160–2.639). In addition, comorbidities such as diabetes mellitus, low body mass index, and low serum high-density lipoprotein cholesterol were associated with higher incidence of events.

**Conclusion:**

History of CHD at dialysis initiation was associated with a higher incidence of heart disease events and mortality and all-cause mortality.

**Trial registration:**

UMIN 000007096. Registered 18 January 2012.

**Electronic supplementary material:**

The online version of this article (doi:10.1186/s12882-017-0495-8) contains supplementary material, which is available to authorized users.

## Background

Chronic kidney disease (CKD) eventually progresses to end-stage renal disease, and is also associated with cardiovascular disease (CVD) events and mortality. CKD is an independent risk factor for CVD [1], and many reports have indicated that CKD is associated with the onset of CVD events, including coronary heart disease (CHD), heart failure, cerebrovascular disease, and death [2–7]. The Kidney Disease: Improving Global Outcomes CKD Work Group 2011 clinical practice guideline indicated that as the estimated glomerular filtration rate (eGFR) declines or urinary protein increases, the incidence of CVD-related death increases [8].

Studies have shown that hypertension, dyslipidemia, activation of the renin-angiotensin system, chronic inflammation, oxidative stress, insulin resistance, poor vitamin D status, and increase in uremic toxins such as indoxyl sulfate, asymmetric dimethylarginine, and fibroblast growth factor 23 are associated with the pathogenesis of CVD in patients with CKD [9–15]. eGFR decline may exacerbate the effects of the above-described factors. Moreover, dialysis initiation increases the risk of CVD because of intradialytic hypotension due to ultrafiltration, acidosis, or mineral metabolism disorders.

Several reports previously indicated that CVD leads to poor outcomes in limited to pre-dialysis or maintenance dialysis patients [3, 16–18]. There are few previous studies describing the prognosis of dialysis patients whose baseline was set at the time dialysis was initiated. Therefore, we examined whether the incidence of heart disease events differed between CKD patients with and without a history of CHD at dialysis initiation. In this study, we defined heart disease events and mortality and all-cause mortality as outcomes. We compared the outcomes by using propensity score (PS)-matching.

## Methods

### Subjects

The subjects were patients in whom dialysis had recently been initiated at the 17 centers that participated in the Aichi Cohort Study of Prognosis in Patients Newly Initiated into Dialysis (AICOPP) between October 2011 and September 2013 [19]. Patients who were withdrawn from dialysis while hospitalized, died while hospitalized, or did not agree to be registered were excluded. The multicenter prospective study cohort included 1,524 patients who were at least 20 years old, had CKD, and provided written informed consent. We excluded 9 patients whose outcomes were unknown, as determined by a survey conducted at the end of March 2015. Thus, we enrolled 1,515 subjects in the study. The dataset analyzed in the present study is presented in the Additional file 1.

### Patient characteristics and data at the time dialysis was initiated (baseline)

The baseline was defined as the time at which dialysis was initiated. Body mass index (BMI) was measured at the first dialysis session. Diabetes was defined as a fasting blood glucose level ≥ 126 mg/dL, random blood glucose level ≥ 200 mg/dL, HbA1c (National Glycohemoglobin Standardization Program) level ≥ 6.5%, use of insulin, or use of oral hypoglycemic agents. Medication use referred to the drugs taken at the time of dialysis initiation. Tests were performed using blood samples taken before the first dialysis session. Blood pressure was also measured before the first dialysis session. The following were considered to be heart failure symptoms and findings: (1) dyspnea or orthopnea with hypoxemia; (2) pulmonary congestion or pleural effusion seen on plain chest radiography; and (3) physical findings related to volume excess, such as edema, weight gain, or jugular venous distension.

### Definition of coronary heart disease

We classified patients into 2 groups by history of CHD (i.e., a CHD group and a non-CHD group. A diagnosis of CHD was based on information taken from the medical records. History of CHD was defined as a history of percutaneous coronary artery intervention (PCI) or coronary artery bypass graft (CABG), ischemic change seen on electrocardiogram with symptoms including chest pain on exertion, or positive findings on stress myocardial scintigraphy.

### Survey of events associated with heart disease and survival prognosis

Events associated with heart disease and survival prognosis as of March 31, 2015 were determined by surveying medical records. For patients who were transferred to other institutions, information was obtained by mailing out survey forms.

### Outcomes

The study outcomes included: (1) heart disease events, including onset of acute coronary syndrome, PCI, CABG, hospitalization due to heart failure, or cardiogenic sudden death; (2) mortality due to heart disease; and (3) all-cause mortality.

### Statistical processing

The Easy R program was used for statistical processing [20]. Patient characteristics and baseline data were compared for the two groups using the t-test for continuous variables and Fisher’s exact test for nominal variables. Heart disease events and mortality and all-cause mortality were compared using the log-rank test for Kaplan-Meier curves for the two groups. The PS, which we calculated using logistic regression models, represented the probability that a patient would be assigned to a group with or without a history of CHD. Using a PS-matching procedure, the 2 groups were similarly distributed, indicating that the differences in covariates between the groups were minimized. Moreover, heart disease events and mortality and all-cause mortality were compared for PS-matched patients. Factors contributing to the onset of heart disease events were examined using univariate Cox proportional hazards regression analysis. In addition to a history of CHD, age, gender, and factors that were significant in the univariate analysis (i.e., diabetes mellitus [DM], BMI, eGFR, serum creatinine, high-density lipoprotein [HDL] cholesterol, and use of β-blockers, antiplatelet agents, or statins) served as explanatory variables for the stepwise multivariate Cox proportional hazards analysis. Continuous variables were expressed as the mean and standard deviation, and categorical variables were presented as percentages. *P*-values less than 5% were considered statistically significant.

## Results

### Comparison of patient characteristics and baseline data

Table 1 shows the patient characteristics and baseline data for the 2 groups. Notably, the CHD group had significantly older patients; a lower percentage of females; a higher prevalence of DM; lower blood pressure, eGFR, and serum phosphorus and cholesterol levels; and higher serum creatinine and adjusted calcium levels. The CHD group also had higher usage rates of β-blockers, antiplatelet agents, loop diuretics, and statins. Thirty-seven patients underwent both PCI and CABG, 113 underwent only PCI, and 28 underwent only CABG.

**Table 1:** Baseline characteristics of all patients

Characteristics *n* (1,515)	CHD group *n* (256)	no CHD group *n* (1,259)	*p* value
Age, yrs	72.7 ± 10.0	66.4 ± 13.4	<0.001
Female, %	22.3	34.4	<0.001
Diabetes mellitus, %	64.8	48.2	<0.001
BMI, kg/m^2^	23.0 ± 3.5	23.6 ± 4.5	0.062
SBP, mmHg	143 ± 26	153 ± 26	<0.001
DBP, mmHg	72 ± 14	78 ± 15	<0.001
eGFR, ml/min/1.73 m^2^	6.4 ± 3.1	5.2 ± 1.9	<0.001
Creatinine, mg/dL	7.84 ± 2.51	9.21 ± 3.30	<0.001
BUN, mg/dL	88.3 ± 28.9	92.4 ± 30.7	0.048
Hemoglobin, g/dL	9.4 ± 1.4	9.4 ± 1.6	0.909
Albumin, g/dL	3.15 ± 0.58	3.21 ± 0.60	0.111
Uric acid, mg/dL	8.8 ± 2.7	8.8 ± 2.3	0.894
Potassium, mEq/L	4.5 ± 0.8	4.6 ± 0.8	0.271
Adjusted calcium, mg/dL	8.8 ± 0.9	8.6 ± 1.1	0.023
Phosphorus, mg/dL	5.8 ± 1.5	6.5 ± 1.9	<0.001
LDL-cholesterol, mg/dL	83 ± 32	91 ± 35	0.002
HDL-cholesterol, mg/dL	41 ± 14	45 ± 17	<0.001
Triglyceride, mg/dL	125 ± 95	125 ± 64	0.959
CRP, mg/dL	2.0 ± 3.3	1.8 ± 4.3	0.532
Use of ARBs or ACEIs, %	61.7	60.1	0.674
Use of β-blockers, %	56.3	30.2	<0.001
Use of anti-platelet agents, %	73.8	21.4	<0.001
Use of warfarin, %	12.9	5.8	<0.001
Use of loop diuretics, %	77.3	63.3	<0.001
Use of statins, %	66.4	34.8	<0.001
Use of ESAs, %	84.8	86.1	0.621

Mean ± Standard deviation

*CHD* coronary heart disease, *BMI* body mass index, *SBP* systolic blood pressure, *DBP* diastolic blood pressure, *eGFR* estimated glomerular filtration rate, *BUN* blood urea nitrogen, *LDL* low density lipoprotein, *HDL* high density lipoprotein, *CRP* C-reactive protein, *ARBs* angiotensin receptor blockers, *ESAs* erythropoiesis stimulating agents

### Comparison of heart disease events and mortality

**Fig. 1:**
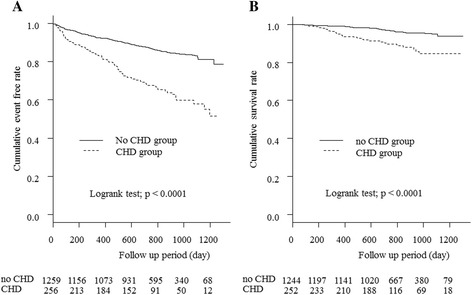
**a** Comparison of events from heart disease between the 2 groups before PS matching. Significant differences were observed between the 2 groups’ cumulative event free rates (*p* < 0.0001). **b** Comparison of death from heart disease between the 2 groups before PS matching. Significant differences were observed between the 2 groups’ cumulative survival rates (*p* < 0.0001). CHD; coronary heart disease, PS; propensity score

Figure 1 displays the Kaplan-Meier curves for the 2 groups in terms of heart disease events and mortality. During observation, heart disease events occurred in 87 patients (34.0%) in the CHD group and 180 (14.3%) in the non-CHD group. The incidence was significantly higher in the CHD group (*p* < 0.0001). Death due to heart disease occurred in 28 patients (10.9%) in the CHD group and 49 (3.9%) in the non-CHD group.

### PS-matched cohort

We performed logistic regression analysis using age, gender, comorbidities such as DM, systolic blood pressure, eGFR, and use of β-blockers and statins to obtain the PS for patients with or without a history of CHD. There were 254 patients in each group. Table 2 shows the patient characteristics and baseline data in the 2 groups after PS-matching. There were no significant differences in variables except for serum HDL cholesterol levels and frequency of antiplatelet agent usage. Table 3 compares the vital signs and cardiac markers at the first dialysis session after PS-matching in the two groups. The CHD group had a significantly higher left ventricular ejection fraction on echocardiography, and higher serum brain natriuretic peptide level.

**Table 2:** Baseline characteristics after PS matching

Characteristics *n* (508)	CHD group *n* (254)	no CHD group *n* (254)	*p* value
Age, yrs	72.7 ± 9.9	73.2 ± 9.3	0.591
Female, %	22.4	26.0	0.406
Diabetes mellitus, %	64.6	63.0	0.712
BMI, kg/m^2^	23.0 ± 3.5	23.3 ± 4.0	0.485
eGFR, ml/min/1.73 m^2^	6.4 ± 3.1	6.0 ± 2.5	0.062
Creatinine, mg/dL	7.85 ± 2.51	8.09 ± 2.45	0.260
BUN, mg/dL	88.2 ± 29.0	88.7 ± 29.1	0.862
Hemoglobin, g/dL	9.4 ± 1.4	9.3 ± 1.5	0.355
Albumin, g/dL	3.14 ± 0.58	3.16 ± 0.56	0.738
Uric acid, mg/dL	8.8 ± 2.7	8.5 ± 2.4	0.146
Potassium, mEq/L	4.5 ± 0.8	4.6 ± 0.9	0.238
Adjusted calcium, mg/dL	8.7 ± 0.9	8.6 ± 1.0	0.193
Phosphorus, mg/dL	5.7 ± 1.5	6.0 ± 1.7	0.050
LDL-cholesterol, mg/dL	83 ± 32	83 ± 33	0.992
HDL-cholesterol, mg/dL	41 ± 14	46 ± 17	0.003
Triglyceride, mg/dL	125 ± 96	111 ± 46	0.059
CRP, mg/dL	2.0 ± 3.3	2.0 ± 4.4	0.946
Use of ARBs or ACEIs, %	61.8	60.2	0.785
Use of β-blockers, %	55.9	48.4	0.109
Use of anti-platelet agents, %	74.0	28.7	<0.001
Use of warfarin, %	13.0	11.0	0.586
Use of loop diuretics, %	77.2	74.0	0.408
Use of statins, %	66.1	63.8	0.642
Use of ESAs	84.6	87.8	0.235

Mean ± Standard deviation

*PS* propensity score, *CHD* coronary heart disease, *BMI* body mass index, *eGFR* estimated glomerular filtration rate, *BUN* blood urea nitrogen, *LDL* low density lipoprotein, *HDL* high density lipoprotein, *CRP* C-reactive protein, *ARBs* angiotensin receptor blockers

**Table 3:** The comparison of vital signs and cardiac markers at first dialysis session between 2 groups

Characteristics *n* (508)	CHD group *n* (254)	no CHD group *n* (254)	*p* value
SBP, mmHg	143 ± 26	145 ± 25	0.462
DBP, mmHg	72 ± 14	72 ± 15	0.579
HR,/min	77 ± 15	76 ± 16	0.778
Heart failure symptom, %	46.5	37.8	0.072
CTR, %	56.6 ± 6.4	56.1 ± 7.1	0.433
LVEF, %	52.9 ± 15.1	62.2 ± 12.0	<0.001
BNP, pg/mL	566 (247, 1345) ^a^	253 (105, 614) ^a^	<0.001
UFV at 1st dialysis session, mL	1,014 ± 960	938 ± 829	0.350

^a^Median (inter-quartile range)

*SBP* systolic blood pressure, *DBP* diastolic blood pressure, *HR* heart rate, *CTR* cardiothoracic ratio, *LVEF* left ventricular ejection fraction, *BNP* brain natriuretic peptide, *UFV* ultrafiltration volume

### Comparison of heart disease events in the two groups after PS-matching

**Fig. 2:**
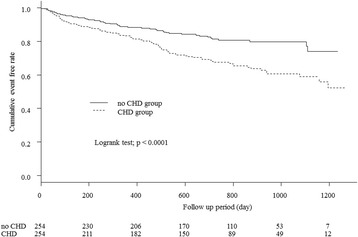
Comparison of death from heart disease between the 2 groups after PS matching. Significant differences were observed between the 2 groups’ cumulative event free rates (*p* < 0.0001). CHD; coronary heart disease, PS; propensity score

Figure 2 displays the Kaplan-Meier curves for the 2 groups after PS-matching, in terms of heart disease events. During observation, heart disease events occurred in 85 patients (33.5%) in the CHD group and 48 (18.9%) in the non-CHD group. The incidence was significantly higher in the CHD group (*p* < 0.0001).

### Comparison of the heart disease-related and all-cause mortality between the two groups after PS-matching

**Fig. 3:**
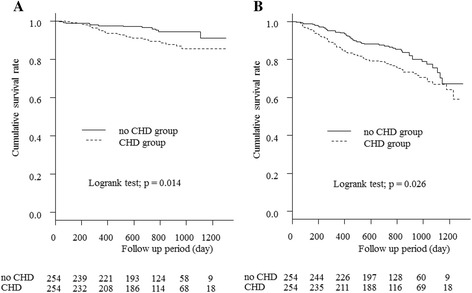
**a** Comparison of death from heart disease between the 2 groups after PS matching. Significant differences were observed between the 2 groups’ cumulative survival rates (*p* = 0.014). **b** Comparison of all-cause death from heart disease between the 2 groups after PS matching. Significant differences were observed between the 2 groups’ cumulative survival rates (*p* = 0.026). CHD; coronary heart disease, PS; propensity score

Figure 3 displays the Kaplan-Meier curves for the 2 groups after PS-matching, in terms of heart disease-related and all-cause mortality. During observation, heart disease-related death occurred in 27 patients (18.9%) in the CHD group and 12 (10.6%) in the non-CHD group. The incidence was significantly higher in the CHD group (*p* = 0.014). All-cause death occurred in 70 patients (27.6%) in the CHD group and 47 (18.5%) in the non-CHD group.

### Factors affecting heart disease events among patients after PS-matching

**Fig. 4:**
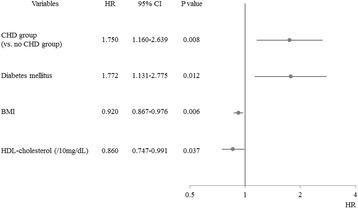
The risk factors for events from heart disease by multivariate Cox proportional hazard analysis using the stepwise method between the 2 groups after PS matching. The CHD group was associated with poorer outcome (HR = 1.750, 95% CI =1.160–2.639, *p* = 0.008). CHD; coronary heart disease, BMI; body mass index, HDL; high density lipoprotein, PS; propensity score, PS; propensity score

Table 4 shows the results of univariate analysis of heart disease events as a response variable. The rate of heart disease events was significantly higher in the CHD group (CHD group vs. non-CHD group: hazard ratio [HR] = 1.955, 95% confidence interval [CI] = 1.369–2.793, *p* < 0.001). In addition, heart disease events were associated with comorbidities such as DM; low BMI; low systolic blood pressure; high eGFR; low serum creatinine; low serum HDL cholesterol; and use of β-blockers, antiplatelet agents, or statins. The results of the stepwise multivariate Cox proportional hazards analysis are shown in Fig. 4. The CHD group had a higher incidence of events associated with heart disease (vs. the non-CHD group, HR = 1.750, 95% CI = 1.160–2.639, *p* = 0.008). In addition, DM, low BMI, and low serum HDL cholesterol level were associated with a higher incidence of these events.

**Table 4:** Hazard ratio (HR) for cardiac disease related events using a univariate Cox proportional analysis in the propensity score-matched cohort

Variables	HR	95% CI	*P* value
CHD group (vs. no CHD group)	1.955	1.369–2.793	<0.001
Age (/10 year)	1.065	0.884–1.283	0.506
Female gender	0.866	0.574–1.308	0.494
Diabetes Mellitus	1.781	1.201–2.641	0.004
BMI	0.949	0.903–0.997	0.036
SBP (/10 mmHg)	0.928	0.867–0.993	0.030
DBP (/10 mmHg)	0.897	0.796–1.012	0.078
eGFR	1.053	1.005–1.104	0.030
Creatinine	0.924	0.860–0.993	0.032
BUN	0.997	0.991–1.003	0.365
Hemoglobin	1.080	0.962–1.212	0.193
Albumin	0.976	0.726–1.313	0.874
Uric acid	1.011	0.944–1.082	0.756
Potassium	0.971	0.793–1.189	0.776
Adjusted calcium	1.113	0.922–1.343	0.265
Phosphorus	0.899	0.805–1.004	0.059
LDL-cholesterol (/10 mg/dL)	0.954	0.897–1.014	0.129
HDL-cholesterol (/10 mg/dL)	0.841	0.735–0.961	0.011
Triglyceride	0.999	0.997–1.002	0.669
CRP	1.022	0.981–1.065	0.292
Use of ARBs or ACEIs	0.895	0.632–1.268	0.532
Use of β-blockers	1.465	1.034–2.075	0.032
Use of anti-platelet agents	1.920	1.344–2.743	<0.001
Use of loop diuretics	1.226	0.808–1.861	0.339
Use of statins	1.756	1.183–2.605	0.005

*HR* hazard ratio, *CI* confidence interval, *CHD* coronary heart disease, *BMI* body mass index, *SBP* systolic blood pressure, *DBP* diastolic blood pressure, *eGFR* estimated glomerular filtration rate, *BUN* blood urea nitrogen, *LDL* low density lipoprotein, *HDL* high density lipoprotein, *CRP* C-reactive protein, *ARBs* angiotensin receptor blockers

## Discussion

In this study, we enrolled patients in whom dialysis was initiated and who were followed thereafter. We confirmed that a history of CHD at the initiation of dialysis was associated with a higher incidence of heart disease events and mortality during dialysis. In addition, the same results were obtained when using PS-matching. We surmised that myocardial remodeling had occurred in these patients. Therefore, disturbed cardiac function can be exacerbated by dialysis initiation, and many factors can arise from dialysis therapy, such as intra-dialysis hypotension due to ultrafiltration. In accordance with the considerations above, the CHD group had poorer prognosis.

We compared the outcomes of the 2 groups using PS-matching. The CHD group had a significantly older age, lower percentage of females, and higher prevalence of DM. The patients in the CHD group were likely to develop these associated events and die of heart disease. Therefore, we used PS-matching to adjust for variables. We confirmed that there was a relationship between history of CHD and outcomes after PS-matching. However, there was no significant difference in the prevalence of heart failure symptoms at dialysis initiation between the 2 groups. We assumed that patients in both groups began dialysis under similar conditions, and that that chronically disturbed cardiac function led to poor outcomes.

Reports of evaluation using the Charlson score for the association between complications or comorbidity before introduction of dialysis, and prognosis after dialysis introduction, have been published sporadically [21–23]. Wu et al. [23] reported the association of a high Charlson comorbidity index with elevated risk of death among nearly 80,000 patients in Taiwan in whom dialysis was initiated. Ivory et al. [24] scored complications and analyzed the relationship of this score to prognosis in more than 2,000 patients in Australia and New Zealand who began dialysis. In their study, the prevalence of CHD as a complication was 39% in the surviving group and 64% in the non-surviving group, which are higher than the rates observed in our study. In the analysis of prognosis, the survival rate at 6 months after dialysis introduction was significantly poorer in the CHD-complicated group (HR = 1.72; 95% CI = 1.51–1.96), similar to our findings. Among the reports of studies confined to CHD, Genesh et al. [25] compared the prognosis in the group receiving hemodialysis treatment with that in the group treated with peritoneal dialysis. They demonstrated that the prognosis after dialysis introduction was poor in patients with a history of CHD, similar to the finding in our study. Patients with a history of CHD accounted for 25.9% of all patients in their study. In our study, the percentage of patients with a history of CHD was only 16.9%, although the mean age was higher in our study than in their study. One factor probably explaining this difference is an ethnic difference. That is, all patients in our study were Japanese, while the percentage of Asians was only 3.7% in the study by Genesh et al. Our study can be characterized by enrollment of only Japanese (i.e., Asian) patients. It is additionally characterized, in comparison to the above-cited studies, by homogeneity of patient background variables (achieved by PS-matching), despite the small sample size. Moreover, in contrast with the above-cited studies in which total deaths were analyzed as the outcome measure, the present study also investigated heart disease events and heart disease-associated death. The results of this study may therefore contain useful information.

A guideline for secondary prevention of myocardial infarction (JCS 2011) recommends the use of antiplatelet agents and statins in patients with CHD [26]. In this study, the usage rates of antiplatelet agents and statins were 73.8 and 66.4%, respectively. We considered that it is difficult to administer antiplatelet agents in some patients with CKD who have a tendency to bleed. On the other hand, administration of statins might have been interrupted because the serum low-density lipoprotein cholesterol levels decreased below the normal range in some patients. This study showed that the CHD group had lower HDL cholesterol levels, even after PS-matching. In addition, a low serum HDL cholesterol level was associated with a higher incidence of heart disease events. Comprehensive management of hypertension, renal anemia, mineral and bone disorders, and dyslipidemia in early stages of CKD before CHD onset could lead to better prognosis. Therefore, more intensive collaboration between nephrologists and cardiologists will be necessary.

The present study has the following limitations. First, we could question the diagnosis of CHD. Although it has the highest reliability for diagnosis, coronary angiography was not used in all patients in the CHD group. Moreover, there are reports describing coronary artery stenosis in asymptomatic patients in whom dialysis was initiated. The results revealed that over 50% of patients had over 75% coronary artery stenosis [27, 28]. Hence, we surmised that there might have been patients in the non-CHD group who had CHD. Acute coronary syndrome or increased severity of CHD due to an increased number of stenotic or occluded coronary arteries is associated with mortality. Second, we could not evaluate the severity of CHD. In addition, the intervals between the onset of CHD and dialysis initiation were inconsistent.

## Conclusion

A history of CHD at the time of dialysis initiation was associated with higher incidence of heart disease events and mortality and all-cause mortality. This observation indicates that CKD patients with CHD should be evaluated more thoroughly.

## Additional file

Additional file 1: The dataset included the patient profiles, comorbidities, medications, and laboratory data at dialysis initiation. (XLSX 202 kb)

## Abbreviations

AICOPP: Aichi Cohort Study of Prognosis in Patients Newly Initiated into Dialysis
BMI: Body mass index
CABG: Coronary artery bypass graft
CHD: Coronary heart disease
CI: Confidence interval
CKD: Chronic kidney disease
CVD: Cardiovascular disease
DM: Diabetes mellitus
eGFR: Estimated glomerular filtration rate
HDL: High density lipoprotein
HR: Hazard ration
PCI: Percutaneous coronary artery intervention
PS: Propensity score
